# Clean hands, safe care: how knowledge, attitude, and practice impact hand hygiene among nurses in Najran, Saudi Arabia

**DOI:** 10.3389/fpubh.2023.1158678

**Published:** 2023-07-13

**Authors:** Awad Mohammed Al-Qahtani

**Affiliations:** Department of Family and Community Medicine, College of Medicine, Najran University, Najran, Saudi Arabia

**Keywords:** hand hygiene, nurses, knowledge, attitude, compliance

## Abstract

**Introduction:**

Healthcare-acquired infections are infections that patients acquire while receiving treatment for a medical or surgical condition and can occur in all care facilities. Hospital-acquired infections and the spread of antimicrobial resistance can be reduced by implementing proper preventive measures, including hand hygiene.

**Aim:**

This study aimed to assess nurses’ knowledge and attitudes toward hand hygiene guidelines in Najran city, determine compliance levels, identify factors contributing to non-compliance, and provide recommendations for interventions to improve hand hygiene practices and reduce healthcare-associated infections risk.

**Subject and methods:**

This cross-sectional study was conducted among nurses working in the selected government hospitals in Najran City, Saudi Arabia. A self-administered questionnaire was distributed among the targeted nurses using an online survey. The questionnaire includes socio-demographic characteristics such as age, gender, and marital status. The questionnaire had 25 items to measure knowledge, 10 to measure attitude, 6 to measure practices, and 4 to measure the impact of COVID-19 on hand hygiene practices.

**Results:**

Among the 386 nurses recruited, 88.3% were females, and 25.6% were aged between 31 to 35 years old. Overall, good knowledge, positive attitude, and good practice levels were found in 42.5, 48.4, and 94%, respectively. The common factor influencing hand hygiene practice was the prevention of cross-infection (88.1%). The total knowledge score mean was 18 ± 3.4 (highest possible score: 25). The total attitude score mean was 37.5 ± 6.1 (highest possible score: 50). The total practice score mean was 26.9 ± 2.8 (highest possible score: 30). A higher score indicates higher KAP of hand hygiene. Factors associated with increased KAP were being older in age (*Z* = 6.446; *p* < 0.001), gender female (*Z* = 9.869; p < 0.001), being a Filipino nurse (*H* = 117.8; *p* < 0.001), working in a surgery department (*H* = 28.37; *p* < 0.001), having more than 10 years of experience (*Z* = 6.903; *p* < 0.001), living in shared accommodation (*H* = 87.22; *p* < 0.001), having associated chronic disease (*Z* = 4.495; *p* < 0.001), and receiving formal training in hand hygiene (*Z* = 2.381; *p* = 0.017). There was a positive highly statistically significant correlation between knowledge score and attitude score (*r* = 0.556), between knowledge score and practices score (*r* = 0.303), and between attitude score and practices score (*r* = 0.481).

**Conclusion:**

In light of the results, we can say that the nurses’ knowledge, attitude, and practice in regards to hand hygiene were deemed acceptable. We noticed that female nurses who were older and had more experience, as well as those who had received formal hand hygiene training, displayed better KAP compared to their counterparts. Moreover, we found a significant and positive correlation between the scores for knowledge, attitude, and practice. Nonetheless, additional research is necessary to establish the extent of KAP concerning hand hygiene.

## Introduction

Patients acquire healthcare-associated infections while receiving treatment for medical or surgical conditions, which can occur in all care facilities (1). Healthcare-associated infections pose serious challenges to the safety of patients and lead to prolongation of hospitalization and utilization of other expensive antibiotics to treat and thus may lead to the emergence of other anti-microbial resistant organisms, disability, and increased cost of care for patients, the economic burden on healthcare systems and higher death rates (2, 3).

A 2-year surveillance study conducted in 14 different types of intensive care units (ICUs) across 106 Ministry of Health hospitals in Saudi Arabia found the central-line associated bloodstream infections rate to be 3.24 per 1,000 central line days, and the overall central line utilization ratio was 0.32 (4). In another study, data from 6,178 patients admitted to ICUs of 12 selected Ministry of Health hospitals were analyzed and reported 13,492 device-associated hospital-acquired infections (5). In 2017, 6 Saudi hospitals conducted a point prevalence survey of inpatients. 6.8% (114/1,666) was the point prevalence. Pneumonia (27.2%), urinary tract (20.2%), and bloodstream (10.5%) infections were most common. 19% of healthcare-associated infections were device-related (6).

However, implementing proper preventive measures, including hand hygiene, can reduce hospital-acquired infections and the spread of antimicrobial resistance. Caregiver’s contaminated hands are an important source of transmission of pathogenic organisms (7–9). Many studies have shown the positive outcome of hand hygiene compliance on incidence of hospital-acquired infections. In a study from Finland, improved compliance with hand hygiene was associated with a decrease in the incidence of hospital-acquired infections. Over the observation period, the number of hospital-acquired infections decreased from 2012 to 1831 and incidence per 1,000 patient days fell from 14.0 to 11.7 (10). In a study from Kuwait, an increased hand hygiene compliance rate led to a significant reduction in hospital-acquired infections per 1,000 patient days, plummeting from 37.2 to 15.1 post-intervention (11). Another longitudinal study carried out in a teaching hospital in China also revealed a decreased incidence of hospital-acquired infections from 1.10 to 0.91% with increased hand hygiene compliance over a period of 4 years (12). A study carried out in King Fahd Hospital of the University, Al-Khobar demonstrated a reduction in the rate of hospital-acquired infections from 3.92 to 2.73 following the implementation of the Six Sigma DMAIC (Define, Measure, Analyze, Improve and Control) model (13).

The 2030 Agenda for Sustainable Development Goals (SDGs and agenda 2030) includes a target related to hand hygiene in healthcare workers. Specifically, the agenda aims to improve access to and utilization of essential medicines and vaccines, as well as strengthen the capacity of healthcare workers in infection prevention and control, including promoting hand hygiene practices. This target acknowledges the critical role that hand hygiene plays in preventing the spread of infections in healthcare settings and underscores the importance of ensuring that healthcare workers have the knowledge, skills, and resources needed to perform effective hand hygiene. Ultimately, achieving this target will contribute to the broader goal of ensuring healthy lives and promoting well-being for all. However, poor compliance with hand hygiene among healthcare workers (HCWs) remains a matter of concern globally. In a study conducted in Argentina, monitored the overall compliance with hand hygiene during routine patient care in intensive care units before and during implementation of a hand hygiene education, training, and performance feedback program. According to the study, there was low hand hygiene compliance. Following the intervention, there was a persistent improvement in hand hygiene compliance, which coincided with a drop in nosocomial infection rates in the ICUs (14). A systematic review conducted by Lambe et al. (15), included 61 studies on hand hygiene compliance, the results revealed the majority were undertaken in high-income countries (60.7%) and in adult intensive care units (85.2%). The average level of hand hygiene compliance was 59.6%. Compliance levels observed to vary by geographic region (high-income nations 64.5%, low-income countries 9.1%), ICU type (neonatal 67.0%, pediatric 41.2%, adult 58.2%), and healthcare personnel type (nursing staff 43.4%, physicians 32.6%, other staff 53.8%). Similarly, studies conducted in Malawi (16), Iran (17), and Saudi Arabia (18) showed insufficient or low compliance rates among HCWs. Nonetheless, compliance with hand hygiene can be improved with multimodal approaches and interventions. Many studies have highlighted the importance of such interventions and the ensuing improvements in compliance with hand hygiene. A multifaceted interventional approach implemented as part of a quality improvement project at the King Abdulziz Medical City intensive care department in Riyadh produced >80% improvement in hand hygiene compliance (19). Similarly, a study carried out among HCWs in ICUs in Aseer Central Hospital, Abha, to assess the level of compliance to hand hygiene practices before and after the multimodal interventional program showed a significant increase in hand hygiene compliance (20).

Nurses comprise the largest share of the healthcare workforce and are critical healthcare team members. Nurses spend significant time in direct contact with patients in caring for them. This puts both patients and nurses at increased risk of hospital-acquired infections if hand hygiene measures are not adhered to (21). In this context, the nurses should have excellent knowledge, a positive attitude and must comply with effective hand hygiene practices as required by the global healthcare bodies. Various studies have assessed nurses’ knowledge, attitude and practice toward hand hygiene with varying degrees of results. In a study in Asir region, 65.4% of the participating nurses achieved good practice scores, while 10.3% achieved inadequate practice scores (22). In a study conducted in primary care settings in Riyadh under the service of King Abdulaziz Medical City, nurses scored 14.1 on the knowledge scale; the maximum achievable score being 21, reflecting a good knowledge score (23). Another study that involved a national sample of HCPs conducted in January–February 2018 gave a broader picture of hand hygiene knowledge, attitude and practices in Saudi Arabia. The average hand hygiene knowledge score was 5.9 (on a 9-point scale), with significant differences across groups. Nurses scored higher than other professionals. The mean hand hygiene knowledge score of HCPs from the Najran region was 6.9. The compliance rate to hand hygiene in the Najran region was 64%, 18th of the 20 studied regions. All the participants showed positive attitudes toward hand hygiene. However, there were only 94 nurses, constituting just 1.3% of the sample, and it was unclear what proportion of specialists or interns were from nursing (24). Another study in Najran Armed Forces Hospital in 2015 evaluated the effectiveness of an interventional program on compliance with hand hygiene among physicians and nurses; however, the sample size was relatively smaller and included just 21 nurses. The intervention positively affected knowledge, attitude and compliance with hand hygiene (25).

The prevalence of hand hygiene knowledge, attitude, and practice among nurses varies across different countries, regions, and healthcare settings. Studies often report a discrepancy between knowledge and practice, with nurses generally having good knowledge and positive attitudes toward hand hygiene, but not always translating that knowledge into consistent practice. The reasons for this discrepancy between knowledge and practice can be attributed to various factors, including workload, inadequate resources, lack of proper training, and organizational culture. According to the WHO, compliance with hand hygiene best practices is only around 9% during care of critically ill patients in low-income countries, and levels of hand hygiene compliance for high-income countries rarely exceed 70%, calling for additional efforts to improve practices all over the world (26). To improve hand hygiene compliance, it is important to address these factors through continuous education, reinforcement of hand hygiene guidelines, provision of necessary resources, and fostering a supportive organizational culture.

Achieving competency in hand hygiene is a crucial aspect of the nursing profession. It is essential to emphasize the importance of hand hygiene among healthcare professionals, especially in light of the rising rates of healthcare-associated infections. Studies have shown that there is room for improvement in the knowledge of and adherence to hand hygiene protocols among nurses. Limited studies have been conducted to identify factors that may influence hand hygiene knowledge and compliance. Considering the significant relevance of the research question in the context of the ongoing COVID-19 pandemic, increasing hospital acquired infections and bacterial resistance, the present study was undertaken to determine nurses’ knowledge, attitude and practice toward hand hygiene in Najran city. The study aims to fill the knowledge gap regarding the hand hygiene practices of nurses. Despite the well-established importance of hand hygiene in preventing healthcare-associated infections, compliance with hand hygiene guidelines remains suboptimal among healthcare workers, including nurses. Hence this study was undertaken with the following objectives: to assess nurses’ knowledge and attitudes toward hand hygiene guidelines in Najran city, determine compliance levels, identify factors contributing to non-compliance, and provide recommendations for interventions to improve hand hygiene practices and reduce healthcare-associated infections risk. By filling this knowledge gap, the study aims to provide insights into interventions that can improve hand hygiene compliance among nurses and ultimately reduce the risk of healthcare-associated infections.

## Methodology

### Study location, sample size and duration of the study

This was a cross-sectional study and included nurses working in the selected government hospitals in Najran city of Saudi Arabia. The study was conducted over a period of 3 months from December 2021 to February 2022. All the nurses who gave consent to take part in the study were included. The study was conducted in Najran University Hospital, King Khalid Hospital, Maternity and Children Hospital, and New Najran General Hospital. In Saudi Arabia, there are now 184,565 registered nurses working in the private sector and other government institutions. The minimal sample size was calculated using the Raosoft sample size calculator^1^ and found to be 384 with a margin of error of 5%, a response distribution rate of 50%, and a confidence level of 95%.

### Development of study tool and its validation

The study tool was developed from the review of relevant literature (27–32). The study tool was mainly based on the questionnaire developed by expert groups from the World Health Organization (27). The modified questionnaire draft was assessed for its content and face validity by experts in the field of infection control. Recommended changes were made, and a language expert reviewed the final manuscript for suitable grammar and usage. To make sure the questions were clear and all ambiguity was avoided, the tool was pilot tested with 20 participants (who were not included in the main sample). In addition, the Cronbach’s alpha was calculated (0.81), demonstrating the reliability of the research instrument.

The knowledge of nurses regarding hand hygiene has been assessed using 25-item questionnaires, where the correct answer for each question has been identified and has been coded with “1” while the incorrect answer has been coded with “0.” The total knowledge has been calculated by summing up all 25 items. A possible score range from 0 to 25 points has been generated. A higher score indicates higher knowledge of HH. By using 50 and 75% as the cutoff points to determine the level of knowledge, nurses were categorized as poor knowledge if the score was below 50, 50 to 75% were categorized as moderate knowledge and above 75% were categorized as good knowledge levels. The cut-off points were selected based on previous studies (28, 29, 32).

Regarding attitude and practices, these had been assessed using 10 items and 6 items, respectively with 5-point Likert scale categories response ranging from “strongly disagree” coded with “1” to “strongly agree” coded with “5.” Negative questions had been re-coded reversely to avoid bias in the score. The total attitude and practices had been calculated by adding all questions, irrespective of each section. The total attitude score has ranged from 10 to 50 points while the total practice score has ranged from 6 to 30 points. A higher attitude and practice score indicates a higher level of attitude and practice toward hand hygiene. Similar to knowledge criteria, 50 and 75% were used as cutoff points to determine the attitude and practice levels.

### Statistical analysis

All data analyzes were performed using the statistical package for social sciences, version 26 (SPSS, Armonk, NY: IBM Corp, United States). Categorical variables were shown as numbers and percentages (%) while continuous variables were summarized as mean and standard deviation. The differences in the scores of knowledge, attitude, and practices, in relation to the socio-demographic characteristics of the nurses were performed using Mann Whitney Z-test as well as Kruskal Wallis H-test. The normality test was carried out using the Shapiro–Wilk test and Kolmogorov–Smirnov test. The KAP scores follow the non-normal distribution. Therefore, the non-parametric tests were applied. Pearson correlation coefficient was carried out to determine the correlation between KAP scores. Two-tailed analyzes with *p* < 0.05 were used as the cutoff for statistical significance while *p* < 0.01 indicates highly statistically significant.

## Results

A total of 386 nurses were recruited. As described in Supplementary Table S1, the most common age group was 31 to 35 years (25.6%) with nearly all nurses being females (88.3%) and 35% being Indian nationality. With regard to education, approximately 70.5% had bachelor’s degrees. 21.2% were working in outpatient clinics and 36.3% had 5 to 10 years of professional experience. With respect to marital status, nearly two-thirds (62.2%) were married and 50.8% were living with their family. The proportion of nurses who were having associated chronic disease was 10.4%. In addition, approximately 90.4% indicated that they received a formal training related to hand hygiene for the last 3 years.

**Figure 1:**
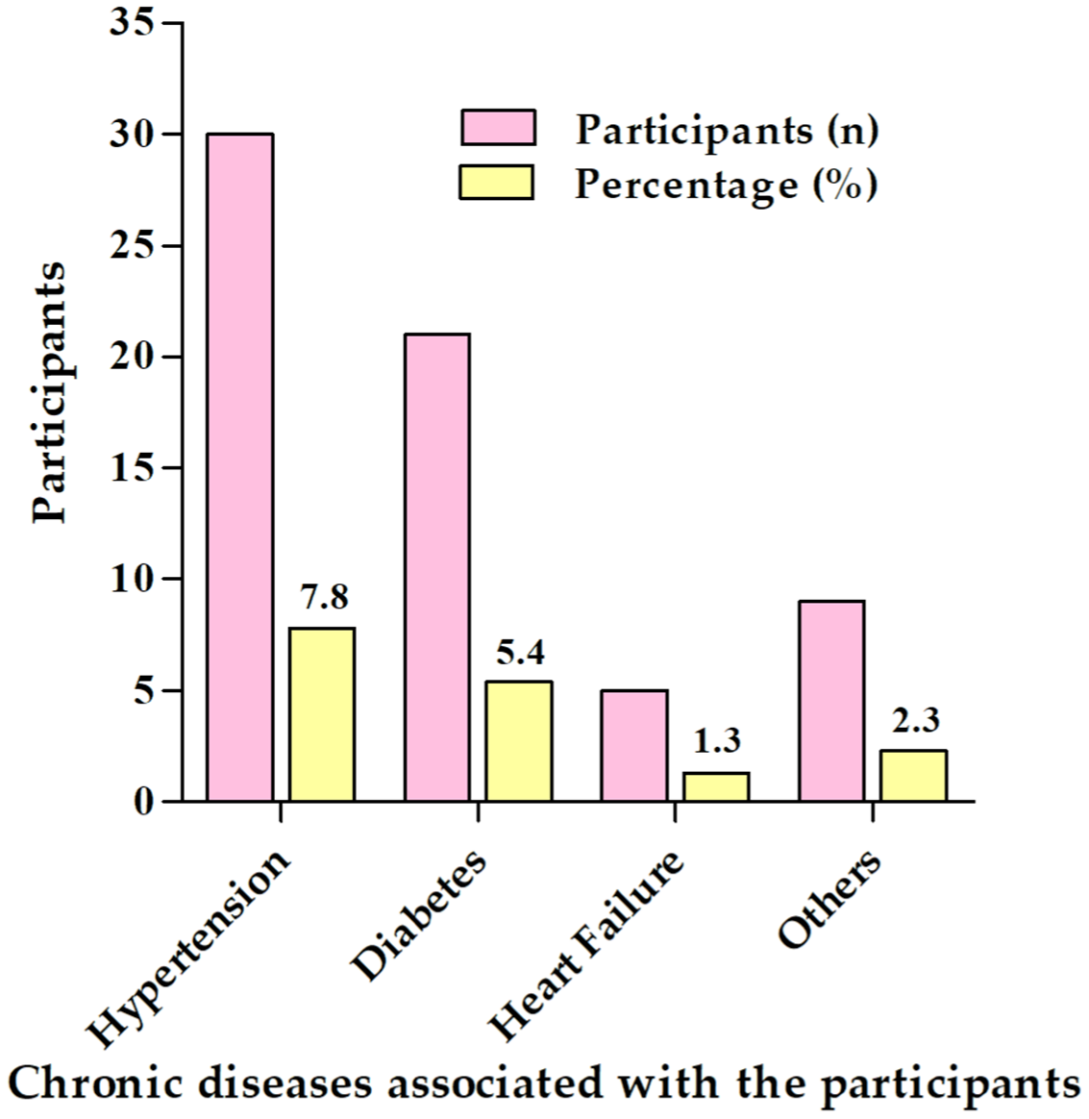
Specific chronic diseases associated with the participants (*n* = 386).

In Figure 1, of those with associated chronic disease, the most common associated chronic disease was hypertension (7.8%) and diabetes (5.4%).

**Figure 2:**
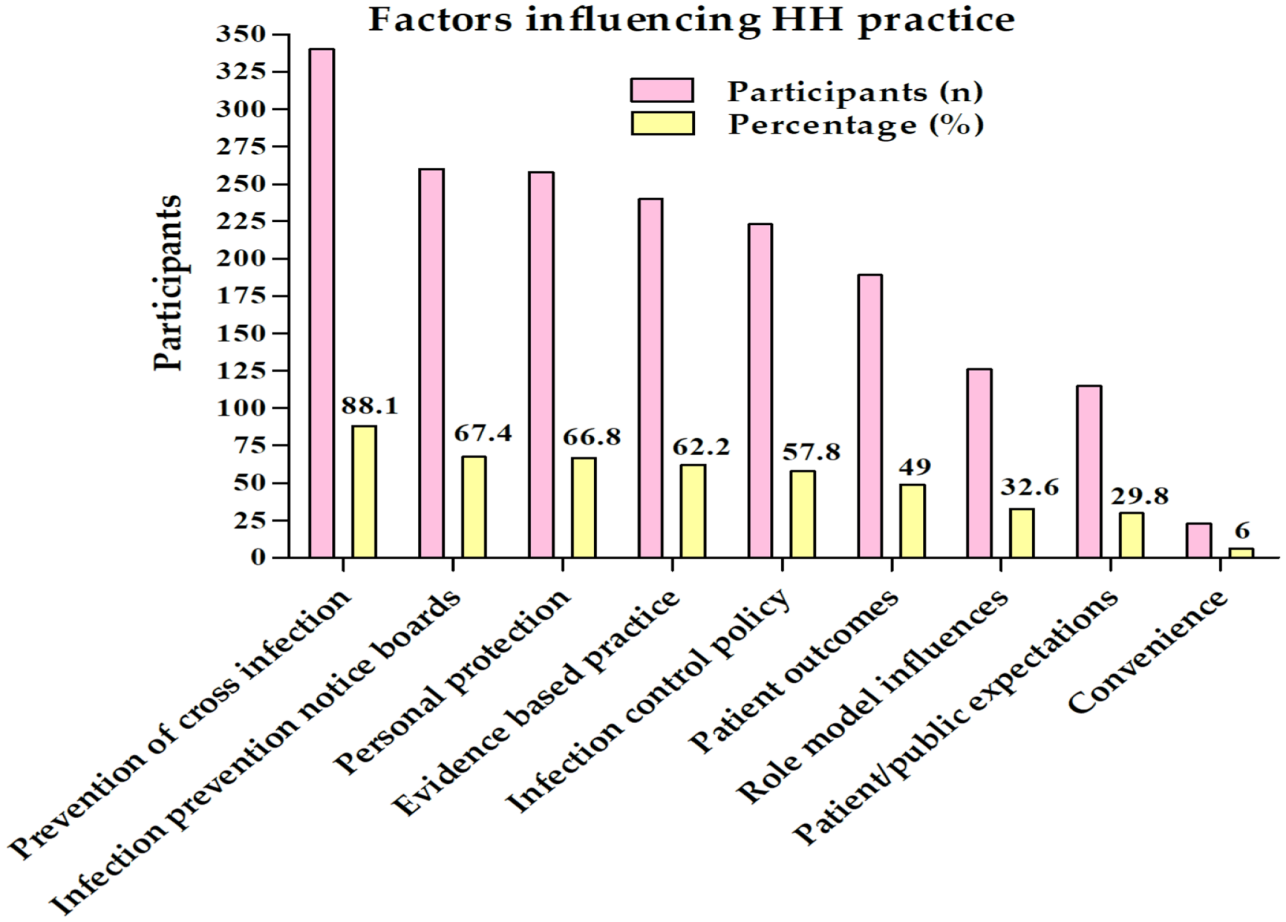
Factor influencing hand hygiene practice among the nurses in Najran, Saudi Arabia (*n* = 386).

In Figure 2, multiple response answers indicated that the common factor influencing hand hygiene practice was the prevention of cross-infection (88.1%), followed by infection of prevention notice board (67.4%) and personal protection (67.4%) while the least of them was convenience (6%).

The assessment of nurses’ knowledge of hand hygiene were given in Table 1. Nearly all nurses (90.2%) were correct that the main route of harmful germs transmission between patients in a healthcare facility was “through healthcare workers’ hands when not clean” while nurses knew that the germs already within the patients were the most prominent source of germs responsible for healthcare-associated infections (64.5%). Also, they were aware of different hand hygiene actions to prevent germs transmission to the patients such as hand hygiene before touching a patient (99%), immediately before a clean/aseptic procedure (94.8%), and immediately after a risk of body fluid exposure (93.3%) whereas 51% were uncertain of the necessity of hand hygiene after exposure to the immediate surroundings of a patient. When asked about the hand hygiene actions to be taken to prevent the transmission of germs to the healthcare worker, almost similar proportions had been noted including hand hygiene after touching a patient (98.2%), immediately after the risk of body fluid exposure (96.1%), and after exposure to the immediate surroundings of patients (95.6%), but nurses were not familiar about the hand hygiene before a clean/aseptic procedure (51.8%). Regarding the pattern of action on alcohol-based hand rub and hand washing, most of the nurses were correct that “hand rubbing is more rapid for hand cleansing than hand washing” (95.3%), however, many of them indicated that the following statements are false including “Hand rubbing is more effective against germs than hand washing (80.1%), “Hand rubbing cases skin dryness more than hand washing” (72.3%) and “Hand washing and hand rubbing are recommended to be performed in sequence” (64.5%). Approximately 70.7% were aware that 20 s is needed for alcohol-based hand rub to kill most germs on hands. 90.9, 80.8, and 74.9% believed that hand rubbing is required before palpation of the abdomen, before giving an injection, and making a patient bend while hand washing is needed after emptying a bedpan (89.9%) and after visible exposure to blood (89.9%). Likewise, 56.7% believed that both hand rubbing and washing are required after removing examination gloves. Approximately 96.9, 92.5, and 85% knew that wearing jewelry, damaged skin, and artificial fingernails can increase the likelihood of colonization of hands with harmful germs while regular use of a hand cream may not (75.1%). Based on the above statements, the overall mean knowledge score was 18 ± 3.4 (highest possible score: 25), with poor, moderate, and good knowledge levels accounting for 10.9, 46.6, and 42.5%, respectively. A higher score indicates higher knowledge of hand hygiene.

**Table 1:** Assessment of nurses’ knowledge of hand hygiene (*n* = 386).

Knowledge statement	*N* (%)
1. Which of the following is the main route of cross-transmission of potentially harmful germs between patients in a healthcare facility? [healthcare workers’ hands when not clean]	348 (90.2%)
2. What is the most frequent source of germs responsible for health-care associated infections? [germs already present on or within the patient]	249 (64.5%)
Which of the following HH actions prevents transmission of germs to the patient?	
3. Before touching a patient [yes]	382 (99.0%)
4. Immediately after a risk of body fluid exposure [yes]	360 (93.3%)
5. After exposure to the immediate surroundings of a patient [no]	197 (51.0%)
6. Immediately before a clean/aseptic procedure [yes]	366 (94.8%)
Which of the following HH actions prevents transmission of germs to the health-care worker?	
7. After touching a patient [yes]	379 (98.2%)
8. Immediately after a risk of body fluid exposure [yes]	371 (96.1%)
9. Immediately before a clean/aseptic procedure [no]	200 (51.8%)
10. After exposure to the immediate surroundings of a patient [yes]	369 (95.6%)
Which of the following statements on alcohol-based hand rub and hand washing with soap and water are true?	
11. Hand rubbing is more rapid for hand cleansing than hand washing [true]	368 (95.3%)
12. Hand rubbing causes skin dryness more than hand washing [false]	279 (72.3%)
13. Hand rubbing is more effective against germs than hand washing [false]	309 (80.1%)
14. Hand washing and hand rubbing are recommended to be performed in sequence [false]	249 (64.5%)
15. What is the minimal time needed for alcohol-based hand rub to kill most germs on your hands? [20 s]	273 (70.7%)
16. Type of HH required before palpation of the abdomen [rubbing]	351 (90.9%)
17. Type of HH required before giving injection [rubbing]	312 (80.8%)
18. Type of HH required after emptying a bedpan [washing]	347 (89.9%)
19. Type of HH required after removing examination gloves [rubbing/washing]	219 (56.7%)
20. Type of HH required after making a patient’s bed [rubbing]	289 (74.9%)
21. Type of HH required after visible exposure to blood [washing]	347 (89.9%)
Which of the following should be avoided, as associated with increased likelihood of colonization of hands with harmful germs?	
22. Wearing jewelry [yes]	374 (96.9%)
23. Damaged skin [yes]	357 (92.5%)
24. Artificial fingernails [yes]	328 (85.0%)
25. Regular use of a hand cream [no]	290 (75.1%)
Total knowledge score (mean ± SD)	18.0 ± 3.40
Level of knowledge	
Poor	42 (10.9%)
Moderate	180 (46.6%)
Good	164 (42.5%)

**Figure 3:**
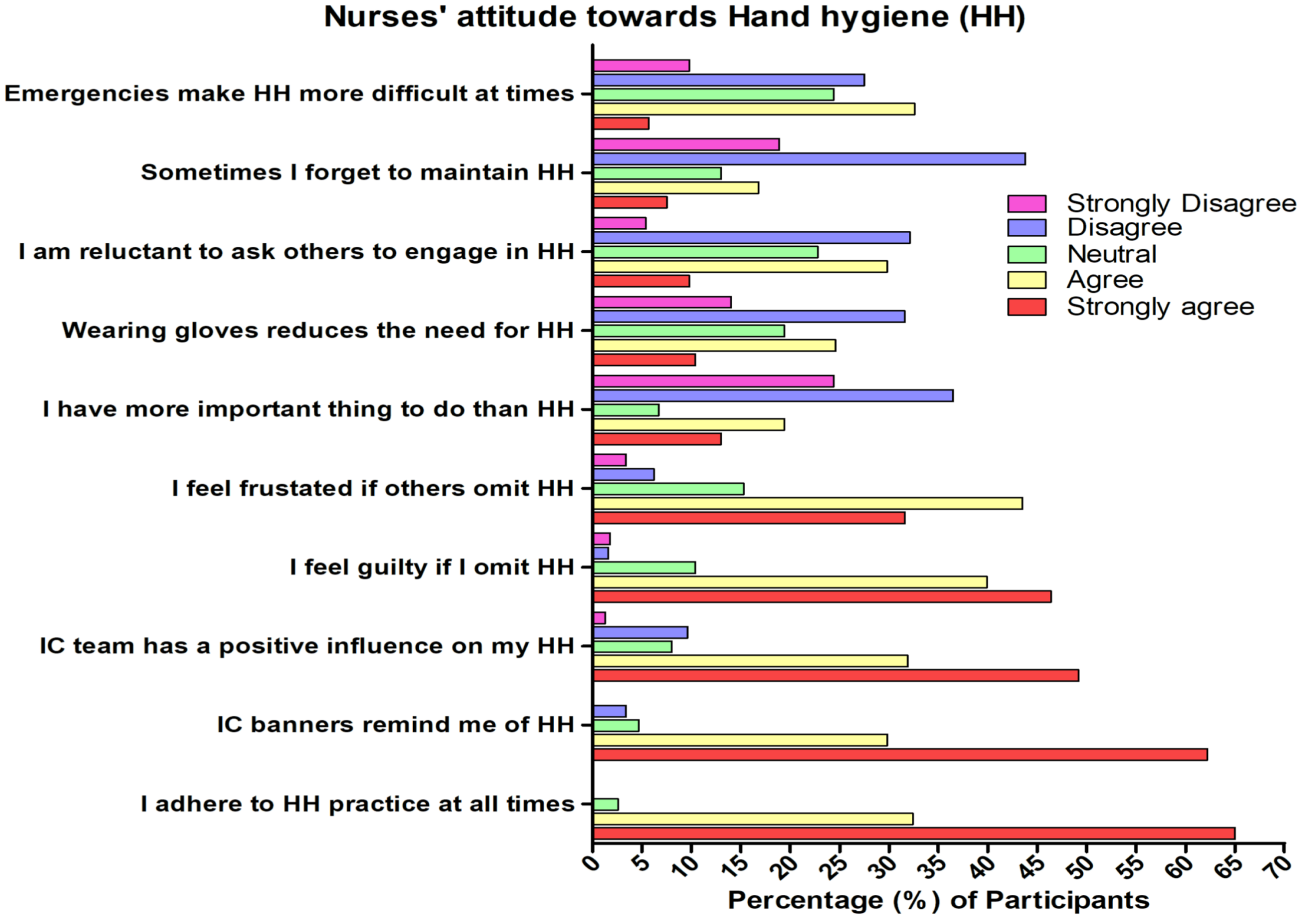
Nurses’ attitude toward hand hygiene in Najran, Saudi Arabia (*n* = 386); HH, Hand hygiene; IC, Infection control.

**Figure 4:**
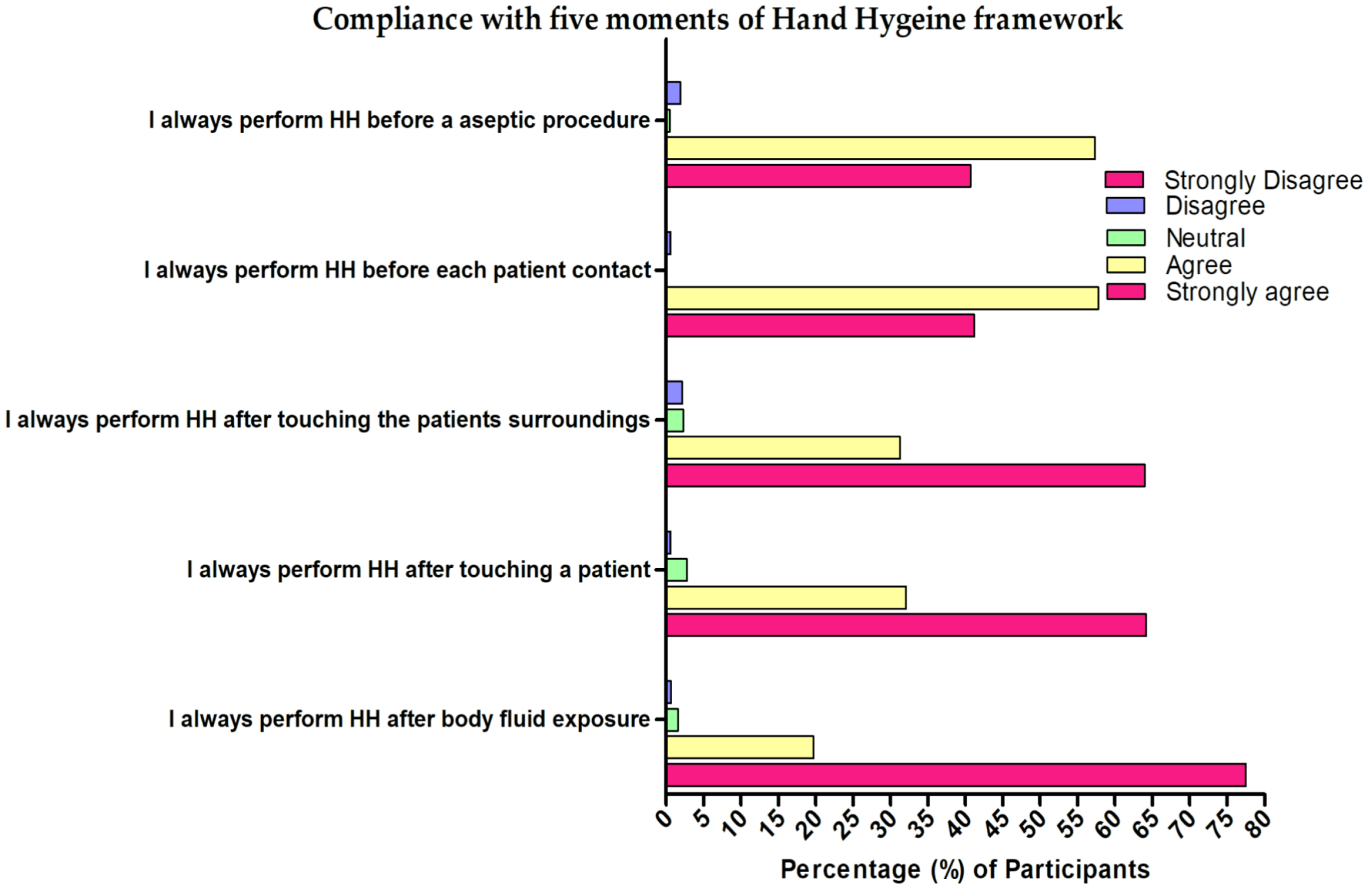
Compliance with five moments of Hand hygiene framework among the nurses in Najran, Saudi Arabia (*n* = 386).

In Table 2, highest attitude ratings were found in the statement “I adhere to hand hygiene practices at all times” (mean score: 4.62), followed by “Infection control banners remind me of hand hygiene” (mean score: 4.51) and “Infection control team has a positive influence on my hand hygiene” (mean score: 4.27). The total mean attitude score was 37.5 (SD 6.11). Positive attitude compromising of 48.4%, neutral 50.8%, and only 0.8% were considered as negative attitude levels. Regarding compliance with the five moments of hand hygiene framework, performing hand hygiene after body fluid exposure showed the highest (mean score: 4.73) while performing hand hygiene before a lean aseptic procedure showed the lowest (mean score: 4.37). The overall mean practices score was 26.9 (SD 2.83) with nearly all considered as having good practices level (94%), 5.2% were moderate and only 0.8% considered to have a poor level of practice (Figures 3, 4).

**Table 2:** Assessment of nurses’ attitudes and practices toward hand hygiene (*n* = 386).

Attitude statement	Mean ± SD
1. I adhere to HH practices at all times	4.62 ± 0.54
2. Infection control banners remind me of HH	4.51 ± 0.74
3. Infection control team has a positive influence on my HH	4.27 ± 085
4. I feel guilty if I omit HH	4.18 ± 1.02
5. I feel frustrated when others omit HH	3.94 ± 1.01
6. Sometimes I have more important things to do than HH^‡^	3.50 ± 1.19
7. Wearing gloves reduces the need for HH^‡^	3.40 ± 1.38
8. I am reluctant to ask others to engage in HH^‡^	3.14 ± 1.23
9. Sometimes I forget to maintain HH^‡^	3.03 ± 1.11
10. Emergencies and other responsibilities make HH more difficult at times^‡^	2.94 ± 1.11
Total attitude score (mean ± SD)	37.5 ± 6.11
Level of attitude	
Negative	03 (0.80%)
Neutral	196 (50.8%)
Positive	187 (48.4%)
Compliance with ‘five moments for HH’ framework	
1. I always perform HH after body fluid exposure risk	4.73 ± 0.58
2. I always perform HH after touching a patient	4.60 ± 0.59
3. I always perform HH after touching a patient’s surroundings	4.57 ± 0.66
4. I always perform HH before each patient contact	4.39 ± 0.55
5. I always perform HH before a clean aseptic procedure	4.37 ± 0.58
6. In your clinical practice, how often (percentage of the time) do you use alcohol-based hand rubs for HH?	4.21 ± 1.00
Total practices score (mean ± SD)	26.9 ± 2.83
Level of practices	
Poor	03 (0.80%)
Moderate	20 (05.2%)
Good	363 (94.0%)

Response has a range from “strongly disagree” coded with 1 to “strongly agree” coded with 5. Practice Q6 has a response range from “never” coded with 1 to “always” coded with 5.

^‡^Reverse coded question.

In Table 3, 73.8% expressed that the pandemic impacted their hand hygiene to a large extent and the crisis brought about a positive change in their attitude toward HH in a similar pattern (77.2%). However, most of them strongly disagreed that there was an increased frequency of alcohol-based hand rub use during COVID-19 than pre-pandemic time (74.4%) and they strongly disagreed that they also increased hand-washing during the COVID-19 pandemic (61.7%).

**Table 3:** Impact of COVID-19 pandemic on hand hygiene practices (*n* = 386).

Statement	*N* (%)
1. Did COVID-19 pandemic impact your HH practices?	
To a large extent	285 (73.8%)
To a moderate extent	67 (17.4%)
To some extent	15 (03.9%)
To a small extent	08 (02.1%)
Not at all	11 (02.8%)
2. Did COVID-19 pandemic bring about a positive change in your attitude toward HH?	
To a large extent	298 (77.2%)
To a moderate extent	61 (15.8%)
To some extent	08 (02.1%)
To a small extent	10 (02.6%)
Not at all	09 (02.3%)
3. Increased frequency of alcohol-based hand rub use during COVID-19 in comparison to pre-pandemic time?	
Strongly agree	0
Agree	04 (01.0%)
Neutral	02 (0.50%)
Disagree	93 (24.1%)
Strongly disagree	287 (74.4%)
4. Increased frequency of hand-washing during COVID-19 in comparison to pre-pandemic time?	
Strongly agree	02 (0.50%)
Agree	11 (02.8%)
Neutral	02 (0.50%)
Disagree	133 (34.5%)
Strongly disagree	238 (61.7%)

In Table 4, there was a positive highly statistically significant correlation between knowledge score and attitude score (*r* = 0.556), between knowledge score and practices score (r = 0.303), and between attitude score and practices score (*r* = 0.481).

**Table 4:** Correlation between the knowledge, attitude and practices scores (*n* = 386).

KAP	Knowledge	Attitude	Practices
Knowledge	1		
Attitude	0.556**	1	
Practices	0.303**	0.481**	1

**Correlation is significant at the 0.01 level (2-tailed).

When measuring the differences in the score of the knowledge, attitude, and practices toward hand hygiene in relation to the socio-demographic characteristics of the nurses (Table 5), it was found that higher knowledge score was more associated with being older (*Z* = 6.446; *p* < 0.001), gender female (*Z* = 9.869; *p* < 0.001), nationality (being a Filipino nurse; *H* = 117.8; p < 0.001), working in a surgery department (*H* = 28.37; *p* < 0.001), having better years of experience (*Z* = 6.903; *p* < 0.001), being married (*Z* = 2.368; *p* = 0.018), living in shared accommodation (*H* = 87.22; *p* < 0.001), having associated chronic disease (*Z* = 4.495; *p* < 0.001) and received formal training in hand hygiene (*Z* = 2.381; *p* = 0.017). Regarding attitude, a higher attitude score was more associated with being older (*Z* = 9.090; *p* < 0.001), gender female (*Z* = 7.682; *p* < 0.001), being a Filipino nurse (*H* = 157.3; *p* < 0.001), being more educated (*Z* = 3.357; *p* = 0.001), working in ICU (*H* = 60.162; *p* < 0.001) having better years of experience (*Z* = 7.640; *p* < 0.001), living in shared accommodation (*H* = 80.398; *p* < 0.001), having associated chronic disease (*Z* = 3.692; *p* < 0.001) and received formal training regarding hand hygiene (*Z* = 4.066; *p* < 0.001). Pertaining to practices, a higher practices score was more associated with older age (*Z* = 8.202; *p* < 0.001), gender female (*Z* = 6.711; *p* < 0.001), being a Filipino nurse (*H* = 50.713; *p* < 0.001) working in ICU (*H* = 33.454; *p* < 0.001), having better years of experience (*Z* = 5.160; *p* < 0.001), being married (*Z* = 2.171; *p* = 0.030), living in shared accommodation (Z = 6.206; *p* = 0.045), having associated chronic disease (*Z* = 2.893; *p* = 0.004) and received formal education regarding hand hygiene (*Z* = 4.776; *p* < 0.001).

**Table 5:** Differences in the score of knowledge, attitude and practices in relation to the socio-demographic characteristics of the nurses (*n* = 386).

Factor	Knowledge score (25) Mean ± SD	Attitude score (50) Mean ± SD	Practices score (30) Mean ± SD
Age group^a^			
≤35 years	16.4 ± 3.94	34.0 ± 5.56	25.3 ± 3.37
>35 years	19.0 ± 2.52	39.7 ± 5.38	27.9 ± 1.84
*Z*-test; *p*-value	6.446; <0.001**	9.090; <0.001**	8.202; <0.001**
Gender^a^			
Male	12.1 ± 2.30	30.9 ± 3.97	23.3 ± 4.43
Female	18.8 ± 2.68	38.4 ± 5.82	27.3 ± 2.14
*Z*-test; *p*-value	9.869; <0.001**	7.682; <0.001**	6.711; <0.001**
Nationality^b^			
Filipino	20.4 ± 1.61	43.4 ± 4.46	28.0 ± 1.69
Indian	17.8 ± 3.03	36.1 ± 5.21	27.3 ± 2.18
Saudi	14.8 ± 3.96	33.1 ± 4.95	24.8 ± 4.03
Other	17.9 ± 2.27	35.7 ± 3.64	26.4 ± 2.44
*H*-test; *p*-value	117.8; <0.001**	157.3 < 0.001**	50.713; <0.001**
Educational level^a^			
Diploma	17.8 ± 2.81	35.5 ± 4.33	26.9 ± 2.33
Bachelor or master	18.1 ± 3.56	38.1 ± 6.42	26.8 ± 2.96
*Z*-test; *p*-value	1.704; 0.088	3.357; 0.001**	0.164; 0.869
Department^b^			
Emergency	17.8 ± 3.72	39.4 ± 7.02	26.5 ± 3.53
Intensive care unit	18.6 ± 3.63	39.9 ± 7.09	27.8 ± 2.73
IM/Medical/Rehabilitation	16.4 ± 3.49	34.6 ± 4.75	27.3 ± 2.57
Mixed medical/surgical	17.6 ± 3.15	33.4 ± 4.34	25.5 ± 2.99
Outpatient clinic	17.9 ± 3.39	37.2 ± 4.67	27.4 ± 2.37
Surgery	19.6 ± 1.92	39.7 ± 4.79	26.7 ± 1.66
*H*-test; *p*-value	28.37; <0.001**	60.162; <0.001**	33.454; <0.001**
Professional experience in years^a^			
≤10 years	17.2 ± 3.46	35.7 ± 5.36	26.3 ± 2.03
>10 years	19.4 ± 2.81	40.7 ± 6.04	27.8 ± 2.15
*Z*-test; *p*-value	6.903; <0.001**	7.640; <0.001**	5.160; <0.001**
Marital status^a^			
Unmarried	17.9 ± 4.28	38.1 ± 7.63	26.2 ± 3.69
Married	18.0 ± 2.74	37.2 ± 4.96	27.3 ± 2.03
*Z*-test; *p*-value	2.368; 0.018**	0.840; 0.401	2.171; 0.030**
Living condition^b^			
Living with family	16.8 ± 3.58	35.6 ± 5.33	26.4 ± 3.31
Shared accommodation	19.8 ± 2.44	40.8 ± 5.95	27.4 ± 2.13
Single accommodation/Living alone	16.7 ± 2.44	33.2 ± 3.41	26.8 ± 2.17
*H*-test; *p*-value	87.22; <0.001**	80.398; <0.001**	6.206; 0.045**
Associated chronic disease^a^			
Yes	19.9 ± 2.59	41.3 ± 7.08	27.9 ± 2.11
No	17.8 ± 3.42	37.1 ± 5.85	26.7 ± 2.88
*Z*-test; *p*-value	4.495; <0.001**	3.692; <0.001**	2.893; 0.004**
Received formal training in HH^a^			
Yes	18.1 ± 3.37	37.9 ± 6.17	27.2 ± 2.43
No	16.9 ± 3.53	33.8 ± 4.07	24.2 ± 4.51
*Z*-test; *p*-value	2.381; 0.017**	4.066; <0.001**	4.776; <0.001**

^a^*p*-value has been calculated using Mann Whitney *Z*-test. ^b^*p*-value has been calculated using Kruskal Wallis *H*-test. **Significant at *p* < 0.05 level.

## Discussion

This study assessed nurses’ knowledge, attitude, and practices regarding hand hygiene in major hospitals in Najran City, Saudi Arabia. The present study highlights nurses’ perspectives regarding hand hygiene in the context of the COVID-19 outbreak. The prevalence of healthcare-associated infections is a significant concern for public health, leading to higher morbidity, mortality, and added expenses within the healthcare system. Maintaining proper hand hygiene is a crucial measure in preventing the transmission of such infectious diseases.

### Knowledge about hand hygiene among nurses in Najran city

In the current study, nurses’ knowledge regarding hand hygiene was satisfactory. Approximately 46.6% were considered to have moderate knowledge, 42.5% were considered good, and only 10.9% were estimated to have poor knowledge levels. A previous study conducted among Saudi nursing students documented a moderate knowledge of hand hygiene (28). Similarly, a study conducted among final year medical and nursing students at the University of Sri Jayewardenepura, Sri Lankan, revealed that the participants had moderate knowledge, but attitudes, and practices of all the participants was overall poor (33). Similarly, in recent studies conducted among healthcare workers in Lahore, Pakistan, revealed moderate knowledge of hand hygiene (34); a study conducted in Iran University of Medical Sciences, Iran (35), showed that majority of the healthcare workers had good knowledge of hand hygiene; a study conducted in Riyadh, Saudi Arabia revealed good to moderate hand hygiene practices (36).

However, in a study conducted in India (29), the knowledge of the nursing and medical students was suboptimal, with only 9% being considered as having a good level of knowledge and that the nursing students exhibited significantly better hand hygiene knowledge than medical students. The study emphasized the importance of training sessions being conducted more frequently, especially among medical students, with continuous surveillance to ensure that the students adhere to the correct hand hygiene practices. A previous Saudi Arabian study conducted in 2016 among Saudi nursing students, demonstrated moderate knowledge and attitude toward hand hygiene (28). Given the present study’s findings, we can infer that there has been an increase in hand hygiene knowledge, attitudes, and practices in Saudi Arabia in recent years. This increase can be attributed to the Ministry of Health policy initiatives, which are focused on providing better health services that are easily accessible and meet high-quality standards.

Several socio-demographic variables were significantly associated with knowledge, including age, gender, nationality, education, department, professional years of experience, marital status, living condition, associated chronic disease and participation in formal training related to hand hygiene. These results seem better than Goodarzi et al. (35). According to their report, although they found a significant relationship between knowledge and education, however, the rest of the socio-demographic variables were not relevant to knowledge, such as age, gender, work experience, ward of the workplace, working shift, and attending the hand hygiene training class in the last 2 years. Similarly, Bimerew and Muhawenimana (37), found no significant factors between knowledge and socio-demographic factors.

In the specific assessment of nurses’ knowledge of hand hygiene, the nurses in the present study demonstrated a good understanding of hand hygiene basic facts. For instance, most nurses knew the main route of cross-transmission of potentially harmful germs (90.2%). They were highly aware of the different actions to be taken to prevent the transmission of germs to the patients and co-healthcare workers. However, they were less likely to be aware that hand hygiene necessitates after exposure to the immediate surroundings of a patient (51%) or immediately before a clean/aseptic procedure (51.8%). Furthermore, the nurses showed a better perception of alcohol-based hand rubs and hand washing with soap and water as they could identify whether the statement was true or false and that most nurses knew the different hand hygiene techniques in the critical area of the hospital settings. In addition, most of the nurses knew the factors that should be avoided to decrease the likelihood of harmful germs, such as wearing jewelry (96.9%), damaged skin (92.5%), and artificial fingernails (85%), but not the use of hand cream (75.1%). Similar results were seen in a study conducted by Zia et al. (34) among healthcare workers in Pakistan. Accordingly, they reported that 76.1% of the healthcare workers agreed regarding the routine use of alcohol-based hand rub, while 81.9% among doctors; however, nurses (70.1%) had more interest in attending formal training about hand hygiene than doctors (44.8%). In our study, nearly all (90.4%) nurses had attended formal training in hand hygiene for the last 3 years, which was higher compared to the study by Zia et al. (34). One of the reasons for better hand hygiene knowledge among healthcare workers in Saudi Arabia is strong emphasis on infection control in healthcare settings by the Ministry of Health. Healthcare workers are trained in infection control measures, including hand hygiene, and are expected to follow strict protocols to prevent the spread of infections. Any knowledge gap in hand hygiene could severely affect patient safety, including COVID-19 transmission. Yadav and Giri (38) observed gaps in hand hygiene knowledge, whereas, Clack et al. (39), emphasized the importance of knowing about patients, HCWs, and the hospital environment, which all contribute to the pathogen reservoir in the clinical setting. Apisarnthanarak and Weber (40), reported that pathogens, including multidrug-resistant strains, can cross-contaminate by being carried into the hands of those in the healthcare environment (40). Kingston et al. (41) emphasized that nurses have a moral, ethical, and professional obligation to follow standard guidelines for optimal hand-washing practice while providing care. Most importantly, nurses should be aware that the hospital environment can contaminate both their hands and their patients (40). Defeating multidrug-resistant organisms primarily depends on HCWs improving their compliance with proper hand hygiene (41).

### Attitude about hand hygiene among nurses in Najran city

The results of the present study indicate a modest attitude among the nurses. Data indicates that 50.8% had a neutral attitude, 48.4% were positive, and only 0.8% showed a negative attitude. These results mirrored the study of Cruz and Bashtawi (28). According to their reports, 52.1% of nursing students had moderate attitudes, while 13.1% demonstrated poor attitude levels. However, in Sri Lanka, a study by Ariyarathne et al. (33) found that medical and nursing students exhibited poor attitudes toward hand hygiene (33).

In the current study, positive attitudes were more prevalent in older nurses, who were of Filipino nationality, had better education, worked in ICU, had better years of experience, living in shared accommodation, had associated chronic disease, and received formal hand hygiene training. One possible reason for the prevalence of hand hygiene practices among Filipino nurses is their strong cultural emphasis on cleanliness and hygiene. In the Philippines, personal hygiene is considered a virtue, and cleanliness is seen as a reflection of one’s character. This cultural emphasis on cleanliness may contribute to a greater awareness and adoption of hand hygiene practices among Filipino nurses.

Hand hygiene practices among nurses working in ICU and those with more experience may be influenced by several factors, including education and training, professional experience, and exposure to infectious agents. Nurses working in ICU are typically highly trained in infection prevention and control measures, including hand hygiene. They work with critically ill patients who are often more susceptible to infections, and they are trained to follow strict protocols to prevent the spread of infections. As a result, they may be more aware of the importance of hand hygiene and may be more likely to practice it consistently. In addition, nurses with more experience may have a greater understanding of the importance of hand hygiene in preventing the spread of infections. They may have had more exposure to infectious agents and may have seen firsthand the consequences of poor hand hygiene practices. As a result, they may be more likely to adopt and consistently practice good hand hygiene habits. Finally, nurses working in ICU and those with more experience may be more familiar with the specific risks and challenges associated with their work environment. For example, ICU nurses may be more aware of the risks associated with invasive procedures and may be more likely to practice hand hygiene before and after these procedures.

In a study conducted in Shaqra University, Cruz et al. (42), reported that female students demonstrated a better attitude toward hand hygiene and had a higher self-reported performance related to the five moments of hand hygiene. In the present study, female nurses also showed significantly higher attitudes than male nurses, consistent with the previous reports. Nurses’ knowledge about hand hygiene mirrors their attitude toward it. Most of the nurses agreed (32.4%) or strongly agreed (65%) to adhere to hand hygiene practices at all times wherein infection control banners remind them of hand hygiene (agree: 29.8%; strongly agree: 62.2%) and have a positive influence on their compliance to hand hygiene (agree: 39.9%; strongly agree: 46.4%). Hence, most get bothered when someone does not comply with hand hygiene (agree: 43.5%; strongly agree: 31.6%).

On the other hand, the nurses tend to strongly disagree (24.4%) or disagree (36.5%) that hand hygiene is not necessary when wearing gloves, and approximately 46% agreed or strongly agreed that it is not easy to ask others to comply with hand hygiene. There have been indications that the nurses showed conflicting decisions, which may have been the barriers toward hand hygiene, such as emergencies and other responsibilities, lack of time, other priorities, and forgetfulness. Among Ireland’s medical and nursing students, Kingston et al. (30) reported that students believe alcohol-based hand rub can cause skin damage, accounting for 52.8% and that adherence to alcohol-based hand rub could lead to dermatological issues. The students cited skin sensitivity (32.5%) and skin damage (19.6%) as the most commonly cited barriers to alcohol-based hand rub. Nurses’ dislike toward hand sanitizers due to skin damage has been reported to impede their adherence to hand hygiene, posing a potential health risk to them and their patients.

### Practice about hand hygiene among nurses in Najran city

In the current study, the results showed that nurses had very good hand hygiene practices. Nearly all (94%) nurses were considered to have good practices, and the rest were either moderate or poor. The results are higher than that of Zia et al. (34), wherein doctors and nurses were considered to have moderate practice levels but comparing the level of practice between doctors and nurses, they discovered that doctors exhibited better hand hygiene practices than the nurses did, while in South Africa (37), the level of practice was suboptimal, specifically between males and females which did not coincide with our results. Another study conducted among nurses working in hospitals in the Asir region of Saudi Arabia revealed that two-thirds of the participants followed a good hand hygiene practice, which was significantly higher among female participants than male participants (43). These results corroborate our study’s findings, which reported a higher KAP in female nurses than their male counterparts.

Hand hygiene practices among female nurses may be more prevalent than male nurses due to several factors, including cultural norms, education and training, and personal attitudes toward hand hygiene. In many cultures, women are traditionally associated with household cleanliness and hygiene. As a result, female nurses may be more likely to adopt and practice good hand hygiene habits. Conversely, male nurses may not have the same cultural expectations or norms associated with cleanliness and hygiene, which may contribute to lower rates of hand hygiene practices. Education and training may also play a role in the prevalence of hand hygiene practices among female nurses. Nursing education programs often emphasize the importance of infection prevention and control, including hand hygiene, and female nurses may be more likely to adhere to these guidelines. Finally, personal attitudes toward hand hygiene may also contribute to the gender differences in hand hygiene practices among nurses, female nurses may have a greater concern for patient safety and a greater sense of responsibility toward their patients, which may lead to more consistent hand hygiene practices.

In the present study, older nurses, female gender, Filipino nationality, working in ICU, having better years of experience, being married, living in shared accommodation, having an associated chronic disease, and receiving formal education in hand hygiene were more likely to exhibit better practices compared to other nurses. These findings may have been similar to Cruz and Bashtawi (28), who found that having a good attitude toward hand hygiene, being aware that hand hygiene is an effective intervention in preventing hospital-acquired infections, participation in hand hygiene training and seminars, and being in a lower academic level of nursing education were identified as predictors of better hand hygiene practice, while in a study by Humran and Alahmary (44), no significant differences in compliance with hand hygiene between students in all categories were reported.

### Compliance with the five moments of the hand hygiene framework

Nurses showed excellent compliance concerning the five moments of the hand hygiene framework. In a 5-point Likert scale category, the ratings are at an all-time high in performing hand hygiene after body fluid exposure risk, hand hygiene after touching a patient, hand hygiene after touching a patient’s surroundings, hand hygiene before each patient contact, and hand hygiene before a clean aseptic procedure, while the use of alcohol-based hand rub for hand hygiene was also seen as high. The practices of the nurses in the five moments of the hand hygiene framework are better than the nursing students in Ireland (30). According to reports, nursing students had suboptimal practices on the framework, with first-year students being more compliant than fourth-year students. They further indicated that 16% of students were unaware of the clinical contraindications for using an alcohol-based hand rub and 9% did not know when to use soap and water or alcohol-based hand rub. In a previous Saudi Arabian study conducted in 2006, although hand hygiene was practiced by 70% of medical students, 18.8% of nurses, and 9.1% of senior medical professionals, the methods used were all unsatisfactory (36). There has to be an in-service educational intervention focused on hand hygiene for nurses and other HCPs. Research has shown that various factors, such as the environmental context, social pressure, and individual attitudes, can impact the practice of hand hygiene. It is our understanding that focusing on beliefs, attitudes, capacity, and supportive infrastructures is crucial in implementing strategies to improve healthcare workers’ knowledge, attitudes, and practices regarding hand hygiene. There are several possible reasons for better hand hygiene knowledge and practice among healthcare workers in Saudi Arabia, which are outlined below:

#### Cultural beliefs

The culture in Saudi Arabia places a high value on cleanliness and hygiene, which may lead to better hand hygiene practices among healthcare workers.

#### Availability of resources

Saudi Arabia has invested heavily in healthcare infrastructure and resources, including hand hygiene products such as soap, water, and hand sanitizers. This availability of resources may make it easier for healthcare workers to practice good hand hygiene.

#### Regulatory requirements

The Saudi Ministry of Health has established guidelines and regulations for infection control in healthcare settings, including hand hygiene. Healthcare facilities are required to comply with these regulations, which may lead to better hand hygiene practices among healthcare workers.

#### Education and training

Healthcare workers in Saudi Arabia receive extensive education and training on hand hygiene and infection control measures. This education and training may help to reinforce the importance of good hand hygiene practices and encourage healthcare workers to follow them consistently.

### Correlation between knowledge, attitudes and practice

A positive and highly statistically significant correlation existed between the knowledge, attitude and practice scores and vice versa. This indicates that the increase in the score of knowledge is correlated with the increase in the score of attitude and practices. These trends are almost in accordance with the study of Zia et al. (34), wherein they found a positive correlation between knowledge and practice; however, a negative correlation was observed between knowledge and attitude, which could be a turning point. Similarly, Goodarzi et al. (35) noted a significant positive correlation between perception and attitude toward hand hygiene.

### Impact of COVID-19 pandemic on hand hygiene practices

Regarding the impact of the COVID-19 pandemic on nurses’ hand hygiene practices, it was observed that despite the significant impact of the pandemic on hand hygiene practices, it brought about a positive change in the nurses’ attitudes to a great extent; however, most of the nurses indicated that they did not change the frequency of hand washing or alcohol-based hand rub use during the pandemic. This is consistent with the study of Sandbøl et al. (45), wherein the COVID-19 pandemic did not raise compliance with hand hygiene among healthcare workers. While in a rapid review and meta-analysis done in China by Wang et al. (46), reported that during the COVID-19 pandemic, there has been a noticeable increase in the adherence of healthcare providers to proper hand hygiene protocols.

### Policy and research implications

This study has significantly contributed to the Kingdom’s limited body of literature on hand hygiene. The Saudi National Transition Program, one of the executive programs to realize Saudi Vision 2030, was created to identify the difficulties government agencies confront and improve the institutional capacity required to fulfill the ambitious vision objectives (47). A patient-centered healthcare system has been implemented by the Ministry of Health through the current strategic plan, with one of the top priorities being curbing the spread of infectious diseases. Saudi Arabia is transforming various sectors as part of its national vision for “Vision 2030,” which includes the healthcare sector. During this transition, it prepared services and supplies for the COVID-19 pandemic before the first case was reported on 2nd March 2020 and implemented public health and social measures, including appropriate hand hygiene practices to control the spread of the virus (48).

### Strengths and limitations

This is the first study to analyze nurses’ attitudes toward HH in the Najran province. The study employed an optimum sample size, making it possible to minimize the danger of reporting false-positive or false-negative results. Our research has some limitations. Since all of our participants were nurses employed by the government sector, our findings do not reflect the KAP of nurses employed by private hospitals and primary healthcare facilities. The study solely included nurses and no other healthcare workers. Furthermore, the study was limited to Najran City, so the findings cannot be extrapolated to the entire province/country. Because the study included several questions that required retrieving knowledge, recall bias cannot be ruled out, and there may be social desirability bias.

## Conclusion

In conclusion, our study highlights the positive state of hand hygiene knowledge, attitude, and practices among nurses working at major hospitals in Najran City, Saudi Arabia. Our findings suggest that older female nurses with more years of experience and those who participated in formal hand hygiene training demonstrated better hand hygiene KAP. To maintain and update the nurses’ knowledge, attitude, and compliance with hand hygiene guidelines, we recommend continuous education through lectures, courses, and symposiums. Additionally, it is important to identify the factors that negatively influence attitudes toward hand hygiene and address them to improve hand hygiene practices in healthcare. Our study provides potential interventions that could enhance hand hygiene practices and lower the occurrence of hospital-acquired infections. These include implementing educational initiatives, enhancing the availability of hand hygiene resources, and creating a healthcare setting that encourages a culture of hand hygiene. Overall, our findings have practical implications for healthcare administrators and policymakers in Najran City and beyond. By prioritizing hand hygiene education and resources, healthcare facilities can improve patient safety and reduce the spread of healthcare-acquired infections.

## Data availability statement

The original contributions presented in the study are included in the article/Supplementary material, further inquiries can be directed to the corresponding author.

## Ethics statement

The studies involving human participants were reviewed and approved by Scientific Ethical Committee of Najran University (NU/2019/12/4). The patients/participants provided their written informed consent to participate in this study.

## Author contributions

The author confirms being the sole contributor of this work and has approved it for publication.

## Funding

The authors are thankful to the Deanship of Scientific Research, Najran University, Najran, Saudi Arabia, for supporting this research through grant research code NU/RC/MRC/11/3.

## Conflict of interest

The author declares that the research was conducted in the absence of any commercial or financial relationships that could be construed as a potential conflict of interest.

## Publisher’s note

All claims expressed in this article are solely those of the authors and do not necessarily represent those of their affiliated organizations, or those of the publisher, the editors and the reviewers. Any product that may be evaluated in this article, or claim that may be made by its manufacturer, is not guaranteed or endorsed by the publisher.

## Footnotes

^1^ http://www.raosoft.com/samplesize.html
