# Supplementary material for: Clean hands, safe care: how knowledge, attitude, and practice impact hand hygiene among nurses in Najran, Saudi Arabia
Source: Front Public Health. 2023 Jul 13;11:1158678. doi: 10.3389/fpubh.2023.1158678 (PMC10372436; doi:10.3389/fpubh.2023.1158678)
Supplement: Supplementary file 1 [file Table_1.DOCX]

**Table S1: Socio-demographic characteristics of the nurses ^(n=386)^**

| **Study Data** | **N (%)** |
| --- | --- |
| **Age group** |  |
| - 21 - 25 years | 65 (16.8%) |
| - 26 - 30 years | 85 (22.0%) |
| - 31 - 35 years | 99 (25.6%) |
| - 36 - 40 years | 84 (21.8%) |
| - >40 years | 53 (13.7%) |
| **Gender** |  |
| - Male | 45 (11.7%) |
| - Female | 341 (88.3%) |
| **Nationality** |  |
| - Egyptian | 38 (09.8%) |
| - Filipino | 112 (29.0%) |
| - Indian | 135 (35.0%) |
| - Pakistani | 13 (03.4%) |
| - Saudi | 77 (19.9%) |
| - Sudanese | 11 (02.8%) |
| **Educational level** |  |
| - Diploma | 85 (22.0%) |
| - Bachelor | 272 (70.5%) |
| - Master | 29 (07.5%) |
| **Department** |  |
| - Emergency | 79 (20.5%) |
| - Intensive care unit | 60 (15.5%) |
| - Internal medicine/Medical | 41 (10.6%) |
| - Long-term/Rehabilitation | 07 (01.8%) |
| - Mixed medical/surgical | 61 (15.8%) |
| - Outpatient clinic | 82 (21.2%) |
| - Surgery | 56 (14.5%) |
| **Professional experience in years** |  |
| - <5 years | 104 (26.9%) |
| - 5 - 10 years | 140 (36.3%) |
| - 11 - 15 years | 83 (21.5%) |
| - 16 - 20 years | 40 (10.4%) |
| - >20 years | 19 (04.9%) |
| **Marital status** |  |
| - Single | 87 (22.5%) |
| - Married | 240 (62.2%) |
| - Divorced or widowed | 59 (15.3%) |
| Living condition |  |
| - Living with family | 196 (50.8%) |
| - Share accommodation with colleagues/friends | 158 (40.9%) |
| - Single accommodation/Living alone | 32 (08.3%) |
| **Associated chronic disease** |  |
| - Yes | 40 (10.4%) |
| - No | 346 (89.6%) |
| **Did you receive formal training in HH in the last three years?** |  |
| - Yes | 349 (90.4%) |
| - No | 37 (09.6%) |
